# Transcatheter Mitral Valve Chordal Repair: Current Indications and Future Perspectives

**DOI:** 10.3389/fcvm.2019.00128

**Published:** 2019-09-04

**Authors:** Alessandro Fiocco, Matteo Nadali, Giovanni Speziali, Andrea Colli

**Affiliations:** ^1^Cardiac Surgery Unit, Department of Cardiac, Thoracic and Vascular Sciences, University of Padua, Padua, Italy; ^2^Division of Cardiac Surgery, St. Louis University, Saint Louis, MO, United States; ^3^Cardiac Surgery Unit, Cardiac, Thoracic, and Vascular Department, University of Pisa, Pisa, Italy

**Keywords:** transcatheter mitral valve repair technologies, mitral regurgitation, transcatheter mitral chordal repair, transcatheter chordal implantation, transcatheter off-pump beating heart neochordae implantation

## Abstract

Transcatheter Mitral Valve Repair (TMVRe) technologies constitute a rapidly expanding field, and have the potential of being adopted as a valuable alternative to surgery in selected patients. TMVRe devices can be distinguished depending on the targeted part of the Mitral Valve (MV) apparatus. Standard classification includes leaflet repair, direct/indirect annuloplasty, chordal repair, and ventricular/chamber remodeling devices. We present the current device situation on chordal repair technologies. Nowadays, transapical off-pump beating heart chordal implantation procedure has become a safe and reproducible option for Degenerative Mitral Regurgitation (DMR). Besides the truly minimally-invasiveness of the procedure, another unique advantage offered by a beating-heart chordal implantation is the real-time assessment of chordal length adjustment during heart cycle with a normally filled left ventricle. Currently, one system is commercially available in Europe, the NeoChord DS 1000 (NeoChord, Inc., St. Louis Park, MN) and the Harpoon TDS-5 (Edwards Lifesciences, Irvine, CA) should become available soon. There is also a diffuse and strong interest to move from a transapical procedure toward a fully transcatheter (transfemoral and transeptal) procedure as shown by the increased number of preclinical programs under development. Interestingly, to achieve outcomes that equate to those of open surgery in DMR, transcatheter therapies will need to follow rigid indications due to strict patient selection criteria for each device, or adopt multiple techniques in a single repair procedure for complex MV disease. Continuous analysis of current clinical results together with future dedicated trial will be of extreme importance to foster the new and upcoming field of transcatheter MV therapy technology development.

## Introduction

Mitral regurgitation (MR) is the most frequent valvular heart disease (VHD) requiring surgery in the United States and the second most common in Europe (1). Currently, surgical mitral valve repair (MVRe) and replacement (MVR) are the treatment options for the management of patients with mitral valve (MV) disease. American and European guidelines support surgical repair over replacement (2) because of the improved survival in degenerative MV disease. In case of functional MV diseases the recommendation is not clear because of presence of conflicting data.

In general, principles of MVRe include preservation or restoration of leaflet anatomy, creation of a large surface of leaflet coaptation and remodeling of the annulus to provide an optimal and stable orifice area.

In recent years, the classical leaflet resection techniques have been challenged by the introduction of more tissue-sparing approaches which include the implantation of artificial ePTFE chords to restore physiological leaflet behavior.

The next step in the progress cycle, which is currently underway, is to move from surgical on-pump procedure toward beating-heart transcatheter solutions (Table 1).

**Table 1:** Table of the present chordal repair technologies.

**Device name**	**Mechanism**	**Trial status**	**Access**	**Figure**
**NeoChord DS 1000** (NeoChord Inc.)	Transapical off-pump beating heart neochordae implantation	CE mark approved	TA	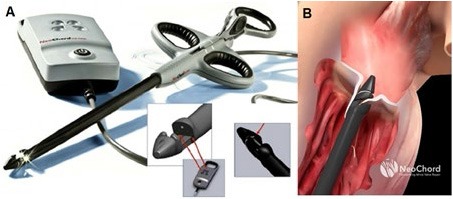
**Harpoon TDS-5** (Edwards Lifescences)	Transapical off-pump beating heart neochordae implantation	Clinical trial completed, commercialization expected for Q1 2020	TA	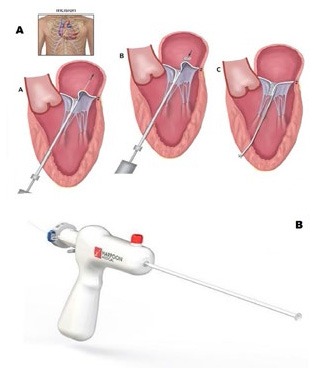
**MitralStitch** (Hangzhou DeJin Medtech Co)	Transapical off-pump beating heart neochordae implantation	Clinical Trial evaluation	TA	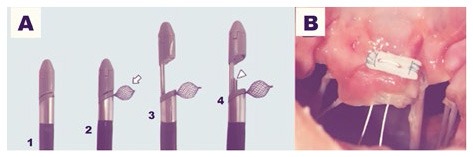
**ChordArt** (Coremedic)	On pump beating heart Neochords sutureless implantation	Clinical Trial (Surgical) Preclinical (TF)	Surgical minithoracotomy (TF under development)	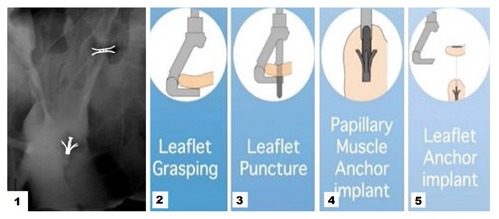
**Valtech V-Chordal Transfemoral** (Valtech)	Trans-septal device for off-pump beating heart neochord implantation	Preclinical underway Clinical Trial (Surgical)	TF	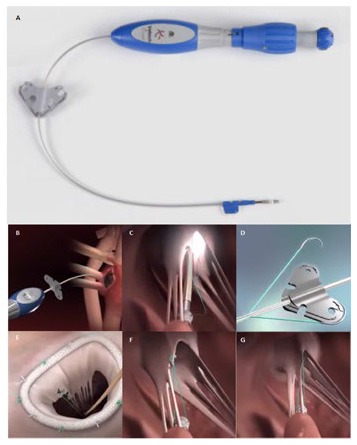
**Pipeline**	Trans-septal device for off-pump beating heart Neochord implantation	Preclinical underway	TF	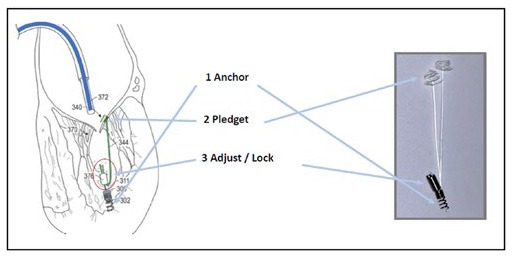
**CardioMech** (CardioMech, Oslo, Norway)	Trans-septal device for off-pump beating heart Neochord implantation	Concept	TF	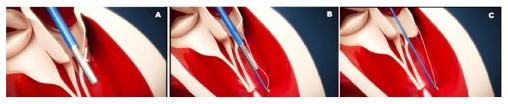
**ChoRe** (ChoRe, Delf, Netherlands)	Trans-septal device for off-pump beating heart Neochord implantation	*Ex-vivo* test	TF	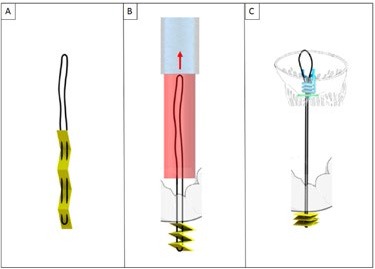
**Mitral Butterfly** (Angel Valve, Vienna)	Trans-septal device for off-pump beating heart Leaflet stabilization and Neochord implantation	Proof of Concept	TF	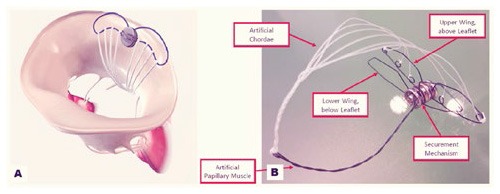

TMVRe devices can be distinguished based on the targeted component of the MV apparatus and classified into leaflet repair, annuloplasty, chordal repair, and ventricular/chamber remodeling. This classification can also include some overlap (3).

Transapical approaches have been recently introduced as well as new transfemoral devices currently in the developing phase.

## NeoChord

The Neochord DS 1000 device (Neochord Inc., St. Louis Park, MN) has been the first transapical chordal implantation device available for clinical use in Europe (4–7). CE mark approval was gained in December 2012 following the results of the TACT trial (4). It is currently under investigation in US, where it is ongoing an IDE trial (RECHORD Trial), comparing surgical MVRe with NeoChord MV repair. An early clinical experience is also growing in Hong-Kong and China with a plan to extend its use to other Asia-Pacific countries in the next future. Currently more than 1,200 patients have been treated with this device.

The procedure is performed under general anesthesia, through a left mini-thoracotomy access in the fifth-intercostal space with 2D/3D real-time TEE guidance. The left ventricle (LV) entry site is identified about 2–4 cm postero-lateral from the real apex in order to obtain a perfect posterior and symmetrical alignment with the papillary muscles (8). LV navigation is performed using 2D X-plane views respecting the standardized step-by-step guide that consider the LV divided in two zones: “chordal free” and “chordal zone” (9). Once the MV plane is crossed, 3D transesophageal echocardiography becomes the leading imaging source. The target portion of the valve is identified, the device is opened and the leaflet is captured using the fiber optic monitor to confirm good leaflet grasp.

**Figure 1:**
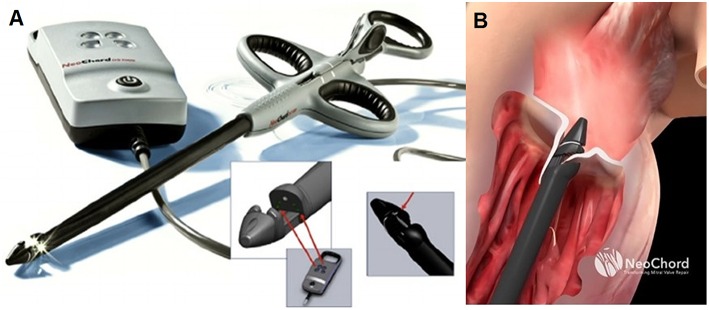
**(A)** Shows the NeoChord DS 1000 system (NeoChord, Inc., St. Louis Park, MN), **(B)** exposes its application on mitral posterior leaflet through a trans-ventricular access.

The leaflet is pierced and the ePTFE chord is passed through the leaflet and retrieved together with the device from the LV. A girth knot is performed allowing for leaflet fixation of the chord. The procedure is repeated for the total number of Neochordae intended to be implanted. Under 2D and 3D TEE control, all the chords are tensioned until adequate leaflet coaptation is achieved, and the neochordae are then secured to the LV wall using a large Teflon pledget (3) (Figure 1).

NeoChord procedural safety has been evaluated by single centers studies showing low mortality (1.4%, 2 extreme-risk patients considered inoperable for conventional surgery) and morbidity, good acute procedural success (98.6%) (10). Patient success (MR ≤ 2+ and freedom from reoperation) was 89% at 1 year (11). A recent multicenter European study, including 213 patients, confirmed high procedural success of 97.6% and good mid-term results with overall survival of 98 ± 1% and patient success of 84 ± 2.5% at 1-year follow-up (5).

More recently Seeburger and coauthors presented the 5 years results of the 6 patients enrolled at University of Leipzig in the TACT trial. Three patients were converted to surgery because of procedural failure and in another 3 patients residual MR at 5 years was significantly less than moderate and experiencing good clinical condition with no symptoms (12).

Based on the initial experience gained by early adopters the procedure underwent continuous technical refinement. LV access site was modified with a more postero-lateral access, echocardiographic views for navigation and grasping were standardized, tensioning was achieved with the use of tourniquets and epicardial stiff Teflon pledget. Moreover, patient selection criteria were refined combining echocardiographic measurement and morphology description.

In particular MV morphology was characterized based on growing complexity as “Type A” isolated central posterior leaflet prolapse/flail, “Type B” posterior multi-segment prolapse/flail, “Type C” anterior or bi-leaflet prolapse/flail, “Type D” para-commissural prolapse/flail or any type of disease with presence of significant leaflet/annular calcifications. Outcomes are strictly connected with the morphological classification (8, 13).

The central echocardiographic selection criteria became the leaflet-to-annulus index (LAI) (14) that evaluate the leaflet to annulus mismatch. LAI is calculated as the ratio between the sum of anterior and posterior leaflet height and the antero-posterior diameter and represents the amount of overriding tissue that could generate the leaflet coaptation surface. The cut-off value of 1.2, corresponding to a 20% excess of leaflet tissue, is significantly related with an MR≤ mild at 1 year follow-up. The LAI can be considered also as an expression of the leaflet-annulus mismatch. The NeoChord ringless procedure showed that annular dilatation should be considered not as an absolute concept but should be always considered in relation to the extension of the leaflets. If LAI is between 1.15 and 1.25 and in presence of isolated central prolapse/flail, a more anterior access site can be achieved in order to improve post-operative mid-term results. This slight modification changes posterior leaflet working angle, stretching it below the anterior leaflet consequently increasing final leaflet coaptation. However, interference with anterior subvalvular apparatus during ventricle navigation should be carefully considered in order to avoid native chordae damage (15).

Acute echocardiographic data analysis, demonstrated a significant reverse remodeling, both of the antero-posterior annular diameter and of the LV cavity volume; these findings were maintained at 1 year follow-up damage (14).

The combination of procedure standardization, technical refinements, and understanding of selection criteria has been analyzed in a single center experience requiring almost 50 procedures as shown by the CUSUM analysis performed (16). Operators had to acquire new surgical skills as well as new visualization training passing from the usual direct surgical view to an echocardiographic real time guidance (17). Despite all these new factors the threshold beyond which the number of deaths or ineffective procedures would be unacceptable was never reached, showing a high safety and efficient profile of the procedure even in its early adoption phase. A recent analysis showed also that the majority of suboptimal results were due to technical factors occurring during the conduct of the operations. To reduce the learning curve effect associated with this procedure, a dedicated preclinical training program was introduced. The training is based on a proctored highly realistic procedural simulation using and *ex-vivo* pulsatile model that reproduces all the steps of the procedure with direct endoscopic and TEE control (18).

Recently, the company announced to be actively involved in the development of a transcatheter chordal repair and edge to edge programs.

In conclusion, Neochord repair procedure is currently considered a viable option for a subset of patients presenting with isolated, simple posterior leaflet lesion set.

## Harpoon

Harpoon Mitral Valve Repair System (MVRS; Edwards Lifescences, Irvine, CA USA) is a sheeted 10 Fr device developed for transapical, beating-heart chord implantation. As for Neochord DS 1000, the procedure is performed under 2D and 3D transesophageal echocardiography guidance. Harpoon system allows the implantation of an ePTFE specially designed chord that is fixed on the MV leaflet by using a preformed double-helix coil knot.

**Figure 2:**
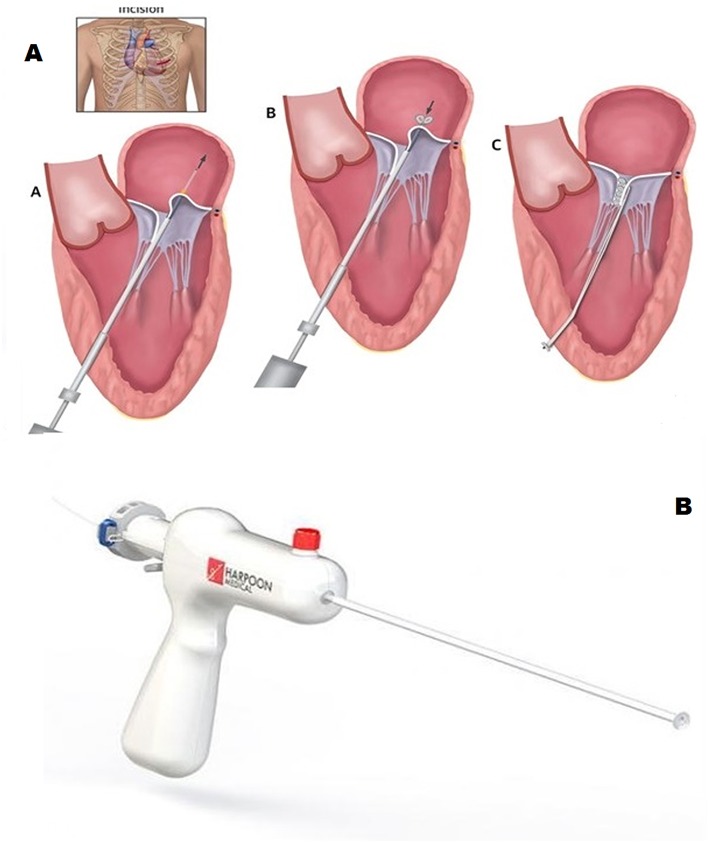
**(A,B)** Show the surgical steps of the chordae implantation with the Harpoon device **(B)**.

Harpoon system is composed of a 14 Fr external diameter introducer with an inner hemostatic valve and a delivery system. The introducer sheet is inserted in the LV more anteriorly than in the Neochord procedure and fixed to the epicardial surface by using conventional “U-pledgets” purse strings. The delivery system contains a 21-gauge needle tightly wrapped with a pre-formed ePTFE bulky knot. When the tip of the delivery system is positioned under the target part of the diseased leaflet using 2D TEE guidance the knot is released by the penetration of the needle through the leaflet tissue. The needle is rapidly withdrawn and the ePTFE coil is tightened forming a double-helix on the atrial surface of the leaflet that secures the artificial chord (19) (Figure 2).

The system is then retrieved from the LV leaving outside the two ends of the ePTFE chord. The procedure can be repeated using a different delivery system for each implant. When the desired number of chords is reached, the introducer is removed and the pure-string sutures closed. Under 2D TEE guidance all the chords are tightened together to reach the desired final coaptation and then are secured to the epicardium with a large Teflon pledget as previously described (20).

Harpoon MVRS received CE mark approval in late 2017 but it is not yet commercially available. The TRACER trial (Mitral TransApical NeoCordal Echo-Guided Repair; prospective non-randomized multicenter clinical study) was conducted to test safety and efficacy of the device. Thirty patients were enrolled in 6 different European Centers. All patients presented severe degenerative MR due to isolated P2 disease. Patient population was highly selected based on presence of an adequate ratio between the posterior prolapse length and the corresponding antero-posterior distance between the free edge of the anterior leaflet and the base of the prolapsed posterior leaflet segment. The ratio should be greater than 1.5, meaning that the redundancy of the tissue should be extremely significant.

Six-month follow-up data were published (21), showing a good safety profile with no perioperative deaths and 20% SAEs rate within 30-days. Procedural success was 93% with two intraoperative conversions to open surgery. At 6-month follow-up, 76% of patients presented mild or less MR, 7% moderate MR, and 7% severe MR and 3 patients underwent conventional reoperation for severe MR recurrence. Moreover, the Harpoon procedure is likely associated to positive LV reverse remodeling with reduction of LV end-diastolic volume at 6-month and with reduction of MV antero-posterior diameter (19% reduction at 30 days, maintained up to 6-month follow-up). More recently, at the 2019 TVT Structural Heart Summit, investigators presented the last updated results of Harpoon one-year follow up clinical experience^1^. Sixty-five patients were enrolled and 62 were treated (1 aborted procedure, 2 converted to open surgery). At follow-up 10 patients exit from analysis because of death (2 cases) or secondary intervention for recurrence of MR (8 cases). With a mean follow-up of 1.4 ± 0.6 years. Of the 52 patients available for echocardiographic evaluation, half presented non/trace MR, 23% mild MR, 23% Moderate MR, and 2% severe MR. Analysis showed a stabilized cardiac revere remodeling observed at 30-postoperative days.

## MitralStitch

**Figure 3:**
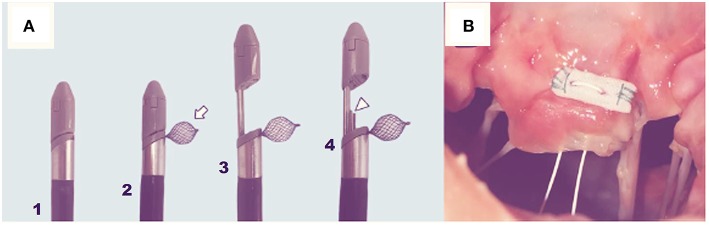
The MitralStich grasping system with confirmation tool highlighted as well as with the nitinol positioner expanded **(A)**. The pledgeted suture deployed on the targeted leaflet **(B)**.

MitralStitch (Hangzhou DeJin Medtech Co Ltd., Hangzhou, China) is a transapical device for beating heart chordal implantation. A pledgeted ePTFE chord is implanted directly in the body of the leaflet avoiding direct suture loop or knots (22). A special feature of the device is the leaflet positioning system, made of a nitinol frame, specifically designed to be retrievable and to provide a precise grasping of the leaflet (Figure 3). The procedure is performed under general anesthesia with echocardiographic guidance through an anterior mini-thoracotomy in the 5th intercostal space. Clinical experience has been evaluated in an early feasibility study that enrolled 10 patients showing 100% procedural success. Recently, investigators showed, as previously reported for the Neochord operation (23), that the same device could be used to perform and edge-to-edge repair implanting chords on both leaflets and by tightening them together with a locking device. A Chinese trial to access market is expected to start in summer 2019.

## ChordArt

**Figure 4:**
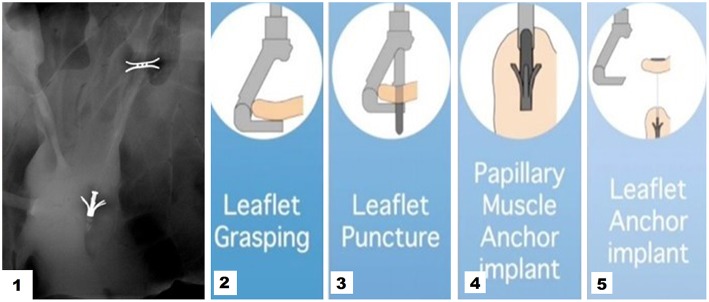
1 Cardiac fluoroscopy showing final ChordArt implantation. 2–5 Implantation steps of the device.

ChordArt (Coremedic, Biel, Switzerland) is a transcatheter mitral repair system. It consists of 3 components: a proximal nickel-titanium anchor for leaflet securement, a distal ventricular/papillary muscle anchor and the ePTFE chord. This special configuration has been proposed to be easily translated into a percutaneous transfemoral-transeptal delivery catheter. The device is currently under clinical evaluation using traditional surgical on pump approach (CHAGALL Trial, NCT03581656). The deployment procedure develops in a stepwise fashion. The leaflet is reached by an antegrade approach and grasped. The leaflet is punctured and the needle tip is passed through the leaflet till the papillary muscle where the first anchoring system is implanted. The needle is then retrieved and the leaflet anchor is released restoring normal leaflet coaptation (Figure 4).

Clinical data of the first surgically implanted device are not yet available.

## V-Chordal

**Figure 5:**
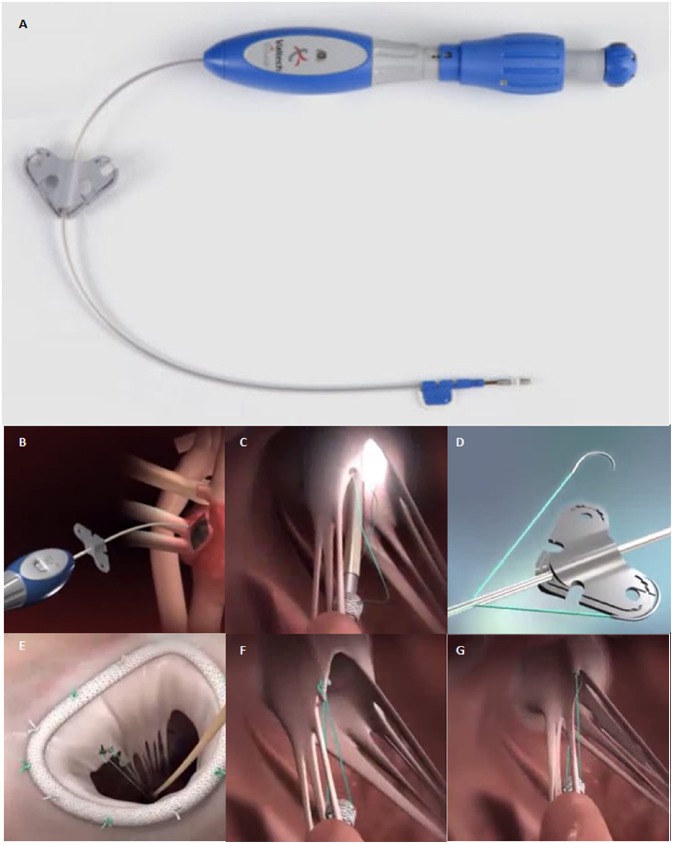
**(A)** The V-Chordal device. **(B)** Crossing of the left atrium and the MV. **(C)** Deployment of the helical fixation anchor on the papillary muscle. **(D)** ePTFE chordal loop is released. **(E)** The new chordae are then sutured to the mitral leaflet. **(F,G)** Final result after chordal length adjustment.

V-Chordal Adjustable Artificial Chordae System (Valtech, Or Yehuda, Israel), is a surgical transcatheter technology allowing on-pump chordal implant with off-pump beating heart length adjustment (24) (Figure 5). Through a left atrial roof incision, the device crosses the left atrium and the MV to reach the papillary muscles where an ePTFE chordal loop is placed. Once helical fixation element have been deployed on papillary muscle, the new chordae are then sutured to the mitral leaflet and atrium is closed leaving the device inside. After weaning from cardiopulmonary-bypass under TEE guidance the surgeon could perform beating heart chordal length adjustment.

Despite clinical-feasibility study has been completed on 6 patients, the transfemoral approach has not been further developed.

## Pipeline

Pipeline (Gore Medical, USA) is a transcatheter device designed for off-pump, beating heart chordal replacement. As seen for other devices, transfemoral-transseptal way is used to access the left atrium and drive a guidewire across the MV inside the LV.

**Figure 6:**
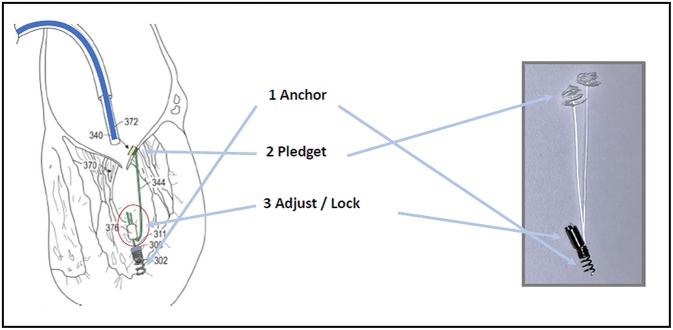
The Pipeline Device.

Once driven on the site, Pipeline is advanced up to the papillary muscles. First, a distal ventricular/papillary muscle helical anchor is deployed. The leaflet is than punctured and the leaflet fixation system is folded up by pulling the artificial Neochord forming this way tissue anchoring. The artificial Neochordae results connected at one side to the ventricle wall by means of the distal anchor and at the other side to the leaflet through the auto-deploying system. The third step consists in suture length adjustment and locking. Under 2D/3D TEE guidance a suture lock device is delivered inside the LV. The chord is then tensioned in order to reach the best coaptation and consequent MR reduction. The residual part of the suture is then cut, disconnecting the device from the LV anchor (Figure 6). The device is under preclinical animal testing.

## CardioMech

CardioMech (Trondheim, Norway) is developing a percutaneous solution for artificial chords implantation. Currently there are no available information about phase of development of the project. The Company website describes a length adjustment device delivered via transvenous-transeptal approach. It comprises a gripper element housing a self-expandable folded anchor made of memory shape material. When the leaflet is grasped, the anchor is unfolded and is secured to the leaflet by piercing it.

**Figure 7:**
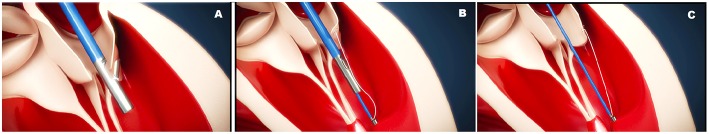
The CardioMech implantation procedure steps. **(A)** PML is grasped and punctured. **(B)** The artificial chord is anchored to the papillary muscle. **(C)** The chord is tensioned.

The catheter device also holds a self-expandable folded papillary anchor made by shape memory metal. The chord extends from the leaflet anchor to the papillary anchor. The chord length is adjustable under real-time echocardiographic guidance. Interestingly the excess of chord length in the atrium is then cut and all catheters are withdrawn (Figure 7).

## ChoRe

**Figure 8:**
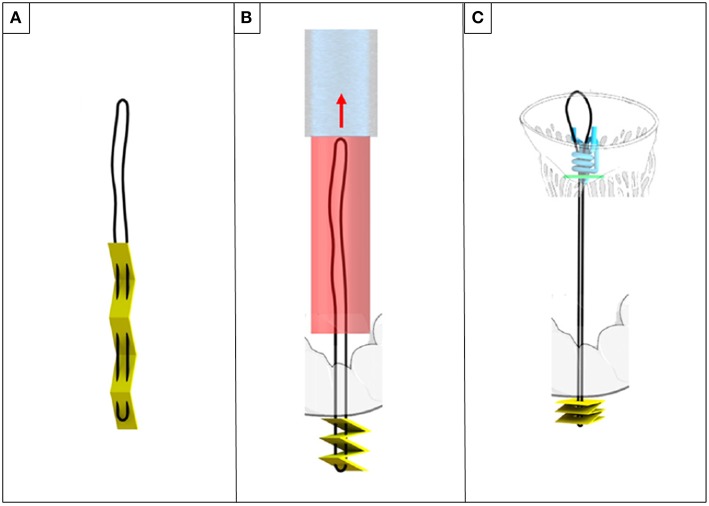
ChoRe **(A)** the artificial chord with the apex pledget. **(B)** The apex pledget folds into an accordion shape, by backword movement of the device and interaction with the ventricle wall. **(C)** The pre-constructed knot is tightened around the chord to provide the correct length and a secure fixation to the atrial wall of the leaflet.

The ChoRe is an underdevelopment device for transfemoral implantation of chords with an epicardial fixation. The procedure steps can be divided in apex fixation, leaflet fixation, and a length adjustment. In the first step of the procedure, a needle puncture the ventricular wall providing externalization of the chords on the epicardium surface together with a folded pledget (Figures 8A,B). In the second step, the device is retrieved toward the mitral valve. Leaflet grasping is performed a needle passes through the leaflet and hooks the implanted chord. The leaflet fixation is achieved with implantation of a pledget. Finally, during the third step, a pre-constructed knot (Figure 8C) is fastened and left on the atrial PML surface.

The system has been recently presented as a concept and *ex-vivo* tests have been performed. A scaled-up size prototype (twice as large as the final device size) has been tested *ex vivo* in bovine hearts (25).

The test showed a good performance of the prototype. Ten artificial chords have been successfully implanted, with an average time of 3.45 ± 1.44 min.

However, the authors disclosed the need for improvement in the apex fixation, leaflet fixation and chordal adjustment phase. In case of apex connection, concerns about bleeding must be considered. Moreover, the length of artificial chords is longer respect to papillary muscle fixation procedures, leading to worse mechanical proprieties of the implant. Finally, the pre-constructed knot seemed not to be able to firmly maintain the point of anchorage when pulled. Future work will focus on improvement of these matters.

## Mitral Butterfly Chordal Mesh Repair

**Figure 9:**
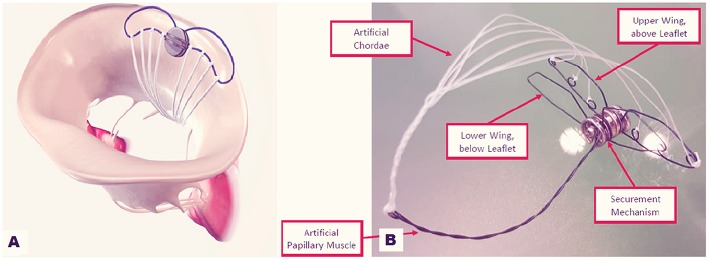
Mitral Butterfly device: **(A)** implanted on the posterior annulus and leaflet. **(B)** The device in its components.

Mitral Butterfly (Angel Valve, Vienna Austria) is a concept technology that can hold and capture the entire prolapsing MV leaflet segment restoring its normal geometry (Figure 9) The device is delivered through a transeptal or transaortic approach and is made of a nitinol-stent with ePTFE yarns which act as artificial chordae. When deployed, the shape memory stent unfolds, holding the prolapsing leaflet segment. The ePTFE yarns replace broken chordae, preventing prolapse/flail. A hook extends in the ventricle, mimicking a papillary muscle, and is coupled with the ePTFE filaments. Thanks to its characteristic design, the mitral annulus remains untouched and no unintentional forces strain the myocardium. Proof of concept was verified using passively perfused porcine hearts. *In vivo* preclinical tests have been planned for 2019.

## Conclusion

The wide variability of MV morphologies drives continuous development of technologies to treat the full spectrum of MR pathophysiology. Consequently a variety of transcatheter MV devices for the percutaneous treatment of MR have been developed. The chordal device spectrum is composed of a big player with a solid clinical experience and many new devices that will enter into clinical practice soon as well as new early phase devices that are still under development. Many companies are working on percutaneous solutions in order to minimize invasiveness as well as to create a more physiological fixation at the level of the papillary muscle or in the base of the LV in between the papillary muscles.

Careful patient selection remains the basic step foregoing any TMVRe technology, and this concept could be even more relevant considering the Chordal Repair therapy. Because of the multifaceted presentation of MV disease, traditional surgical procedures have always combined different leaflet and annular therapies. The current solid surgical background will stimulate the already presented MV transcatheter repair tool-box concept (26, 27) for complementary use of different devices to perform a surgical-like transcatheter MV repair. Moreover, the appropriate combination of leaflet, chordal, ventricular devices together with annular devices and the concomitant innovation in imaging and catheter development will progressively improve long-term outcomes, allowing a future extension of these technology indications to lower-risk patients and the adoption of them as a first-line treatment strategy. However, we must consider that despite early positive results, that are better than those of the early Mitraclip experience and better than what was predicted by the traditional surgical community, long-term durability and effectiveness remain to be proven for all transcatheter chordal devices.

## Author Contributions

All authors listed have made a substantial, direct and intellectual contribution to the work, and approved it for publication.

### Conflict of Interest Statement

AC received travel grants from NeoChord Inc, GS is the inventor of the NeoChord DS 1000 device and is a shareholder of NeoChord Inc. The remaining authors declare that the research was conducted in the absence of any commercial or financial relationships that could be construed as a potential conflict of interest.

## Footnotes

^1^https://www.tctmd.com/slide/harpoon-transapical-technology-and-clinical-updates

## Abbreviations

TMVRe: Transcatheter Mitral Valve Repair
MV: Mitral Valve
MR: Mitral Regurgitation
PML: Posterior Mitral Leaflet
LV: Left Ventricle
VHD: Valvular Heart Disease
DMR: Degenerative Mitral Regurgitation
MVRe: Mitral Valve Repair
MVRS: Mitral Valve Repair System
ePTFE: expanded Polytetrafluoroethylene
TEE: Transesophageal Echocardiography
LAI: Leaflet-to-Annulus Index
TA: Transapical
TF: Transfemoral.
